# A Case of Non-cirrhotic Hyperammonemic Encephalopathy in a Patient With Metastatic Gastrointestinal Stromal Tumor

**DOI:** 10.7759/cureus.37541

**Published:** 2023-04-13

**Authors:** Il Seok D Jeong, Parinaz Abiri, Johnny Cai, Catherine Yim, Leland Powell

**Affiliations:** 1 Internal Medicine, Olive View - University of California, Los Angeles (UCLA) Medical Center, Sylmar, USA; 2 Hematology and Oncology, Olive View - University of California, Los Angeles (UCLA) Medical Center, Sylmar, USA; 3 Neurology/Radiology, Olive View - University of California, Los Angeles (UCLA) Medical Center, Sylmar, USA

**Keywords:** hyperammonemia, sunitinib, gastrointestinal stromal tumor (gist), tyrosine kinase inhibitors (tki), hyperammonemia-encephalopathy, non-cirrhotic hyperammonemia

## Abstract

Acute toxic encephalopathy (ATE) is a widely recognized medical emergency with an expansive differential. One particular known etiology for ATE is elevated ammonia, a powerful neurotoxin that often presents with clinical findings of confusion, disorientation, tremors, and in severe cases, coma and death. Hyperammonemia is most commonly associated with liver disease and presents as hepatic encephalopathy in the setting of decompensated cirrhosis; however, in rare cases, a patient may suffer from non-cirrhotic hyperammonemic encephalopathy. We describe the case of a 61-year-old male with metastatic gastrointestinal stromal tumor who was diagnosed with non-cirrhotic hyperammonemic encephalopathy, and briefly explore the literature describing its mechanisms.

## Introduction

Acute toxic encephalopathy (ATE) is a well-recognized medical emergency encountered in patients with a wide range of medical conditions. Common causes of ATE include metabolic etiologies such as hyperammonemia, endocrinopathies (thyroid dysfunction, hypoglycemia, and adrenal crisis), uremia, electrolyte derangements, vitamin B1 and B12 deficiencies, hypertension (posterior reversible encephalopathy syndrome), and hypoxia-ischemia, as well as infection, intoxication, and vascular disease.

While hyperammonemic ATE is a recognized complication of advanced cirrhosis, hyperammonemia can be encountered in rare cases in the absence of cirrhosis and even in patients without any pre-existing hepatic disease (e.g. primary or metastatic hepatic lesions or portal-venous shunts). While decreased clearance of ammonia is the cause of hyperammonemia in patients with cirrhosis, hyperammonemia may result from decreased clearance or increased production in patients without cirrhosis. If untreated, hyperammonemia can be life-threatening, resulting in encephalopathy, cerebral edema, brain herniation, coma, and death [1].

Due to the aforementioned complications, it is important to consider hyperammonemia even in the absence of cirrhosis. In particular, systemic treatment of cancers using medications such as tyrosine kinase inhibitors (TKIs) may act as a provoking factor. Here, we describe the case of a patient presenting with non-cirrhotic hyperammonemic encephalopathy from metastatic gastrointestinal stromal tumor (GIST). We also discuss the possible association of hyperammonemia with certain tumor types and cancer treatments to raise awareness of an underreported phenomenon.

## Case presentation

A 61-year-old male with a history of metastatic, high-risk GIST with mesenteric, peritoneal, liver, and small bowel masses was evaluated in the hospital for acute onset of confusion, generalized weakness, and somnolence. His diagnosis of metastatic GIST was diagnosed three years prior to his current presentation. At that time, he was started on the first-line treatment with imatinib 400 mg daily, which was followed by dose escalation to 800 mg daily six months after initial diagnosis. Due to the progression of disease, he was transitioned to sunitinib 50 mg daily two years after the diagnosis, which was subsequently dose reduced to 37.5 mg daily within a month of initiation due to grade 3 fatigue, where he remained preceding his presentation. 

**Figure 1:**
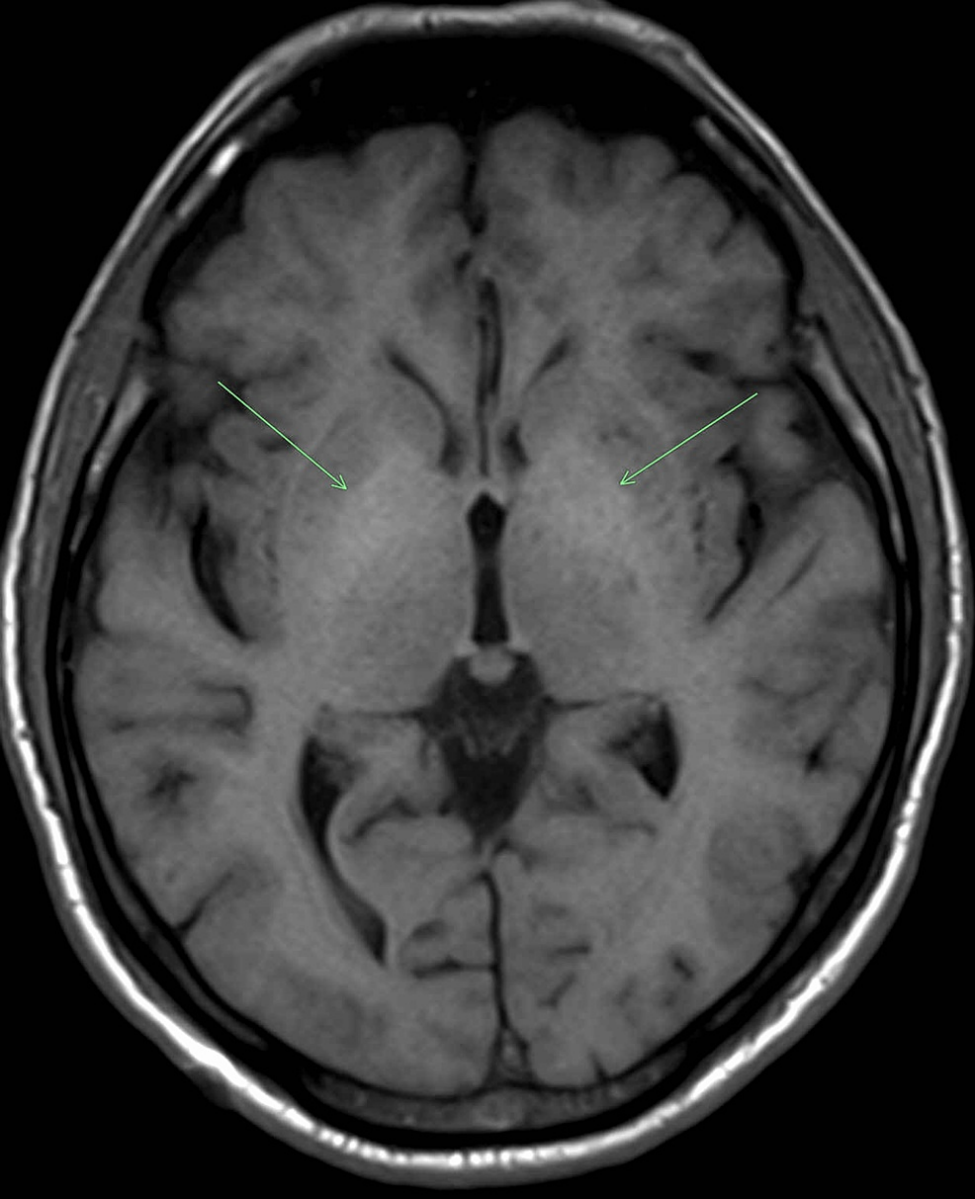
MRI brain in the axial view demonstrating subtle pre-contrast T1 hyperintensity in the bilateral basal ganglia (green arrows).

**Figure 2:**
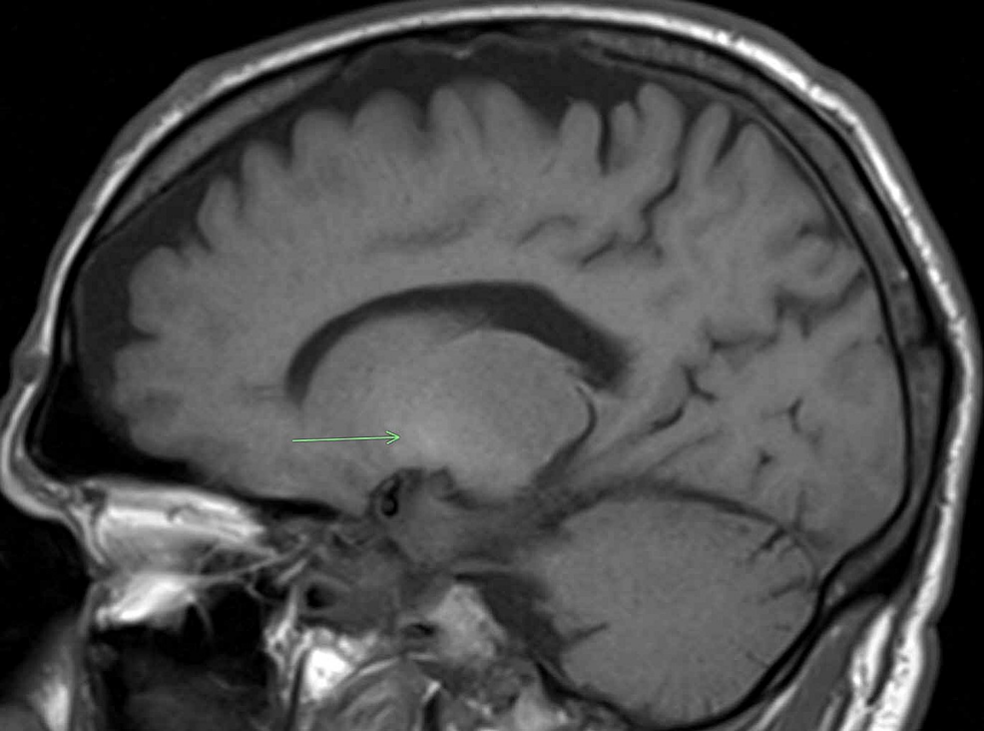
MRI brain in the left sagittal view demonstrating subtle pre-contrast T1 hyperintensity in the left basal ganglia (green arrow).

On presentation, vital signs demonstrated a chronically elevated blood pressure of 160/100. Physical examination demonstrated a lack of orientation to person, place, or time. Neurologic examination demonstrated asterixis. The remainder of the exam did not demonstrate stigmata of chronic liver disease. Table 1 summarizes his laboratory data on admission. Due to his neurologic examination, serum ammonia was obtained and notable for elevated value of 154 µmol/L. HIV, syphilis, blood cultures, and urinalysis were negative. CT head without contrast did not demonstrate acute hemorrhage, midline shift, or edema. Subsequent MRI brain with and without contrast revealed pre-contrast subtle T1 hyperintensity in the bilateral basal ganglia (Figures 1-2), with overlying chronic changes, including an old lacunar infarct in the left para-midline pons belly and findings of old lacunar infarct in the right thalamus. Mild-to-moderate chronic microvascular ischemic changes and scattered old microbleeds in the bilateral cerebral hemispheres were additionally demonstrated. Due to clinical concern for symptomatic hyperammonemic encephalopathy in the absence of clear inciting factors, an aggressive bowel regimen with lactulose with a goal of five to six daily bowel movements was started with the aim of achieving ammonia clearance. Post-treatment serum ammonia levels 24 hours after the start of lactulose demonstrated clearance to 96 µmol/L and correlated well clinically with neurologic recovery to baseline. Due to complete neurologic recovery, our patient was discharged on lactulose with a target of three to four daily bowel movements and re-challenged with sunitinib 37.5 mg at the time of discharge. On the six-month follow-up, he continues to maintain regular bowel movements without further signs and symptoms of encephalopathy. 

**Table 1:** Laboratory studies on admission.

Laboratory Data		Reference Range
WBC (White Blood Count)	5,900 µL	4,500-10,000 µL
Hgb (Hemoglobin)	14 g/dL	13.5-16.5 g/dL
PLT (Platelet)	152,000 µL (Baseline)	160,000-360,000 µL
Na (Sodium)	139 mmol/L	136-144 mmol/L
K (Potassium)	4 mmol/L	3.6-5.1 mmol/L
Cl (Chloride)	107 mmol/L	97-108 mmol/L
Bicarbonate	21 mmol/L	22-32 mmol/L
BUN (Blood Urea Nitrogen)	14 mg/dL	8-20 mg/dL
Creatinine	1.29 mg/dL (Baseline)	0.5-1.2 mg/dL
Glucose	108 mg/dL	65-139 mg/dL
Calcium	10.4 mg/dL (Baseline)	8.9-10.3 mg/dL
INR (International Normalized Ratio)	1.04	0.88-1.14
Albumin	3.9 g/dL	3.5-4.8 g/dL
Alkaline Phosphatase	98 U/L	38-126 U/L
AST (Aspartate Transaminase)	31 U/L	15-41 U/L
ALT (Alanine Transaminase)	24 U/L	14-54 U/L
Total Bilirubin	1.7 mg/dL	0.1-1.2 mg/dL
Direct Bilirubin	0.3 mg/dL	0.1-0.4 mg/dL
Venous pH	7.42	7.33-7.43
Venous Carbon Dioxide	36 mm Hg	38-50 mm Hg
Vitamin B12	445 pg/mL	213-816 pg/mL
TSH (Thyroid-Stimulating Hormone)	2.475 µU/mL	0.35-4.94 µU/mL
Ammonia	154 µmol/L	9-35 µmol/L

## Discussion

Hyperammonemia results as a consequence of either decreased nitrogen clearance or increased nitrogen production. Common causes of decreased nitrogen clearance include portosystemic shunts, inborn errors of metabolism, and medications such as diuretics, barbiturates, anti-epileptic agents (e.g. carbamazepine and valproic acid), narcotics, or corticosteroids [1-2]. Increased nitrogen production can occur via increased muscle metabolism in cases such as trauma, seizure, or starvation. Miscellaneous causes of nitrogen production resulting in hyperammonemia additionally include urease-producing bacterial infections, malignancy, chemotherapeutics (e.g. cytarabine, vincristine, etoposide, L-asparaginase, cyclophosphamide, and 5-fluorouracil), or total parenteral nutrition. Malignancy, in particular, exerts a chronically increased state of protein catabolism and increased susceptibility to other catabolic states such as sepsis and gastrointestinal (GI) bleeding. Case reports have described hyperammonemia in a narrow spectrum of tumor types (e.g. GIST, myeloma, neuroendocrine tumors, hepatocellular carcinoma, renal cell carcinoma, and colorectal carcinoma) [3-14]. GIST in particular is well known for complications related to endophytic growth leading to GI bleeding, obstruction, and perforation. Exophytic growth can cause local compressive effects or infiltration, resulting in failure of involved organs. Interestingly, there has been a case report of non-cirrhotic patient with GIST with the absence of malignant hepatic infiltration presenting with hyperammonemic encephalopathy due to ammonia-producing GIST and concurrent portosystemic shunts into the right renal vein and the right renal cortex, whose encephalopathy resolved with surgical excision of the tumor [3]. 

Targeted therapies and chemotherapeutic agents are also known triggers of encephalopathy. Two well-documented types of adverse events from these agents that present as encephalopathy are posterior reversible encephalopathy syndrome (PRES) and hyperammonemic encephalopathy. Multiple reports of adverse events related to PRES with the use of TKIs such as sorafenib, sunitinib, pazopanib, and cabozantinib have been reported in the literature, with the hypothesis that their anti-angiogenic properties can increase systemic blood pressure [15]. The underlying mechanism of TKI-induced hyperammonemic encephalopathy is poorly understood but has been well-documented across multiple therapeutic agents. Sunitinib, regorafenib, pazopanib, and sorafenib specifically have been linked with hyperammonemic encephalopathy across the aforementioned tumor types in patients with and without chronic liver disease [4]. Proposed mechanisms invoke the principle of TKI-mediated derangement of VEGF and platelet-derived growth factor receptor signaling resulting in damage to the cerebral vascular endothelium. An increase in permeability of the blood-brain barrier ensues, allowing ammonia to easily transverse and cause neuronal dysfunction [16]. There are currently no literature data to explain the mechanism of a TKI-induced direct increase in serum ammonia levels.

Our patient likely had multiple contributing mechanisms that explain his presentation of non-cirrhotic hyperammonemic encephalopathy: first, he had significant malignant infiltration of the liver and associated tumor burden; second, multiple prior cerebral infarcts may have rendered him more susceptible to encephalopathy from subclinical insults; third, sunitinib, the main targeted systemic therapy agent for our patient, may have played a part in further making the patient susceptible to hyperammonemic toxicity. It is noteworthy that our patient was able to continue sunitinib following his hospitalization without relapse in encephalopathy after the start of stable dosing of lactulose. It is difficult to say which of the three aforementioned mechanisms was the trigger to his acute presentation, but these mechanisms likely worked synergistically to produce encephalopathy. 

## Conclusions

While encephalopathy is a commonly presented medical condition, it is important to consider some of its less common causes, especially in the setting of cancer and its treatment. This case report highlights that patients presenting with hyperammonemic encephalopathy may do so in the absence of chronic liver disease. Special attention should be paid in patients with a narrow spectrum of tumor types (e.g. GIST, myeloma, neuroendocrine tumors, hepatocellular carcinoma, renal cell carcinoma, and colorectal carcinoma). Additionally, given continued advances made in TKI therapies, it is important to maintain awareness of their potential to exacerbate encephalopathy, especially in patients with known predisposing co-morbidities.
